# Clinical Outcomes for Metastatic Renal Cell Carcinoma (mRCC) Patients Ineligible for Front-line Clinical Trials

**DOI:** 10.15586/jkcvhl.v11i3.352

**Published:** 2024-08-30

**Authors:** Nathan Reynolds, Wei Wei, Kimberly Maroli, Amanda Bonham, Amanda Nizam, Timothy D. Gilligan, Christopher Wee, Shilpa Gupta, Moshe C. Ornstein

**Affiliations:** 1 Department of Internal Medicine, South Pointe Hospital, Cleveland Clinic, Cleveland, OH, USA; 2 Quantitative Health Sciences, Cleveland Clinic, Cleveland, OH, USA; 3 Department of Hematology and Medical Oncology, Cleveland Clinic Taussig Cancer Institute, Cleveland, OH, USA

**Keywords:** clinical trials, eligibility criteria, immunotherapy, kidney cancer, renal cell carcinoma

## Abstract

Clinical trials for immunotherapy-based regimens in metastatic renal cell carcinoma (mRCC) have extensive inclusion and exclusion criteria. We investigated the clinical outcomes in a real-world cohort of patients who would not have met the criteria for inclusion in front-line mRCC trials. Patients treated with ipilimumab/nivolumab and axitinib/pembrolizumab for front-line mRCC were identified and divided into clinical trial eligible (CTE) and clinical trial ineligible (CTI) cohorts based on key inclusion or exclusion criteria from their respective Phase-3 registration trials. Clinical outcomes were compared in CTE and CTI cohorts. A total of 62 patients treated with axitinib/pembrolizumab and 103 treated with ipilimumab/nivolumab were identified. The International Metastatic RCC Database Consortium (IMDC) criteria were similar across CTE and CTI patients in axitinib/pembrolizumab and ipilimumab/nivolumab cohorts. In the axitinib/pembrolizumab cohort (n = 62), 24 (39%) patients were CTI. The major reasons for the ineligibility were lab abnormalities (n = 11), histology (n = 9), and brain metastases (n = 3). There was no significant difference in response rates (P = 0.08). The median progression-free survival (PFS) was numerically longer in CTE patients (28 vs 12 months; P = 0.09). The overall survival (OS) was higher in the CTE patients (P = 0.02). In the ipilimumab/nivolumab cohort (n = 103), 59 (57%) were CTI. The most common reasons for ineligibility were brain metastases (n = 18), lab abnormalities (n = 16), and histology (n = 16). There was no significant difference in response rates (P = 0.22). However, PFS (P = 0.003) and OS (P < 0.0001) were higher in the CTE patients. In conclusion, many real-world patients are ineligible for RCC clinical trials and had worse outcomes when compared to trial-eligible patients. Additional treatment options are needed for these patients, as well as strategies to include them in prospective trials.

## Introduction

There has been incredible progress in the treatment of patients with metastatic renal cell carcinoma (mRCC). This is largely a result of the introduction of immunotherapy-based combinations for patients with treatment-naïve mRCC. These regimens have a checkpoint inhibitor (CPI) immuno-oncology (IO) agent backbone in combination with either another IO agent (IO/IO) or a vascular endothelial growth factor receptor tyrosine kinase inhibitor (IO/TKI). The FDA has approved five such combinations for patients with treatment-naïve clear cell mRCC. These included one IO/IO combination (ipilimumab/nivolumab) and four IO/TKI combinations (axitinib/pembrolizumab, axitinib/avelumab, cabozantinib/nivolumab, and lenvatinib/pembrolizumab) (1–5).

However, it is well-established that the positive clinical trial results that lead to regulatory approval are not always replicated in real-world clinical practice (6). Several reasons for this phenomenon have been described including, but limited to, issues related to clinical trial design, data interpretation, conflicts of interest, and biological heterogeneity between clinical trials and real-world clinical practice (6, 7). A primary concern often raised about the applicability and relevance of clinical trial results to the broader population relates to the restrictive inclusion and exclusion criteria in clinical trial design that do not reflect the real-world clinical population (8). It is unclear whether these restrictive eligibility criteria are clinically meaningful and impact patient outcomes. This study therefore investigated the clinical outcomes in a real-world cohort of patients who would not have met the criteria for inclusion in front-line mRCC trials.

## Materials and Methods

Patients at Cleveland Clinic, treated with ipilimumab/nivolumab or axitinib/pembrolizumab from July 2014 to February 2021 for front-line mRCC were identified. They were classified as clinical trial eligible (CTE) and clinical trial ineligible (CTI) cohorts based on key inclusion or exclusion criteria from their respective Phase-3 registration trials (CheckMate 214 and KEYNOTE-426) (1, 5). Patient- and disease-specific features were analyzed, and reasons for trial ineligibility were identified.

The summary of patient characteristics was provided in median, IQR, and range for continuous variables, and in frequencies and percentages for categorical variables. The overall survival (OS) and progression-free survival (PFS) were calculated from IO start date to death or last follow-up (LFU) date for OS, and to progression date, death, or LFU date for PFS. Kaplan–Meier method was used to estimate the OS and PFS. Log-rank test was used to compare OS and PFS between patient groups. Statistical analysis was performed using SAS Studio 3.7 (SAS Institute, Cary, NC) and R version 4.2 (R Foundation, Vienna, Austria).

## Results

### Baseline characteristics

A total of 62 patients treated with axitinib/pembrolizumab and 103 treated with ipilimumab/nivolumab were identified. The baseline characteristics were reflective of a typical mRCC cohort (Table 1). In the axitinib/pembrolizumab cohort, most of the patients were men (71%). The median age was 62.6 (40.2–88.3), with 82% clear cell histology, and most (58%) of them had an intermediate risk per the International Metastatic RCC Database Consortium (IMDC) criteria. Similarly, in the ipilimumab/nivolumab cohort, most patients were men (76%). The median age was 62.0 (26.8–87.7), with 80% clear cell histology, and most (58%) of them fell under the category of intermediate risk as per IMDC (Table 1).

**Table 1:** Baseline characteristics.

	Axitinib/Pembrolizumab (n = 62) N (% or range)	Ipilimumab/Nivolumab (n = 103) N (% or range)	Total (n = 165) N (% or range)
Gender			
Male	44 (71)	78 (76)	122 (73)
Female	18 (29)	25 (24)	43 (26)
**Age (median, range)**	62.6 (40.2–88.3)	62.0 (26.8–87.7)	62.3 (26.8–88.3)
Histology			
Clear cell	52 (84)	82 (80)	133 (81)
Non-clear cell	10 (16)	21 (20)	32 (19)
Sarcomatoid features	5 (8)	7 (7)	12 (7)
Prior nephrectomy	41 (66)	59 (57)	100 (61)
IMDC risk			
Favorable	16 (26)	22 (21)	38 (23)
Intermediate	36 (58)	60 (58)	96 (58)
Poor	10 (16)	21 (20)	31 (19)
Sites of metastatic disease			
Lung	40 (65)	66 (64)	106 (64)
Bone	14 (23)	31 (30)	45 (27)
Liver	14 (23)	16 (16)	30 (18)

### Clinical trial eligibility

In the axitinib/pembrolizumab cohort (n = 62), 24 (39%) patients were clinical trial ineligible (CTI). The major reasons for ineligibility were lab abnormalities (n = 11), histology (n = 9), and brain metastases (n = 3). In the ipilimumab/nivolumab cohort (n = 103), 59 (57%) were CTI. The reasons for ineligibility were brain metastases (n = 18), lab abnormalities (n = 16), and histology (n = 16). Additional reasons for clinical trial exclusion are outlined in Table 2.

**Table 2:** Reasons for exclusion from clinical trials.

	Axitinib/Pembrolizumab (n = 62) N (%)	Ipilimumab/Nivolumab (n = 103) N (%)	Total (n = 165) N (%)
Patients who are clinical trial ineligible	24 (39)	59 (57)	83 (50)
Lab abnormalities	11 (46)a	16 (27)b	27 (33)
Histology	9 (38)c	16 (27)d	25 (30)
Brain metastases	3 (13)	18 (31)	21 (25)
Cardio/Vascular	2 (8)e	8 (14)f	10 (12)
Prior or concurrent malignancy/prior systemic therapy	3 (13)	6 (10)	9 (11)
Other	N/A	2 (3)g	2 (2)
Patients with ≥ 1 reason for trial exclusion	4 (17)	7 (12)	11 (13)

a Abnormal renal function (n = 4), anemia (n = 3), elevated liver function tests (n = 2), prolonged INR (n = 1), proteinuria (n = 1).

b Abnormal renal function (n = 13), elevated liver function tests (n = 2), and one patient had both elevated LFTs and thrombocytopenia.

c Unclassified (n = 7), Papillary (n = 1), Chromophobe (n = 1).

d Unclassified (n = 9), Chromophobe (n = 4), Papillary (n = 2), fumarate hydratase (FH)-deficient renal cell carcinoma (n = 1).

e Both patients had prolonged QTc.

f Prolonged QTc (n = 1), poorly controlled hypertension despite appropriate medical therapy (n = 1), contraindicated anticoagulation (n = 5), contraindicated anticoagulation and low ejection fraction (n = 1).

g n = 1 each for KPS < 70, and concomitant use of prednisone 20 mg/day.

There was no difference in clinical trial eligibility based on IMDC risk criteria. Patients who had a prior nephrectomy were more likely to be CTE in both the axitinib/pembrolizumab (P = 0.01) and the ipilimumab/nivolumab (P = 0.04) cohorts. The presence of bone metastasis was associated with CTI status in the axitinib/pembrolizumab cohort (P = 0.03) but not in the ipilimumab/nivolumab patients (P = 0.67). The reverse was observed with liver metastasis, which was associated with CTI status only in the ipilimumab/nivolumab cohort (P = 0.002).

### Outcomes

**Figure 1:**
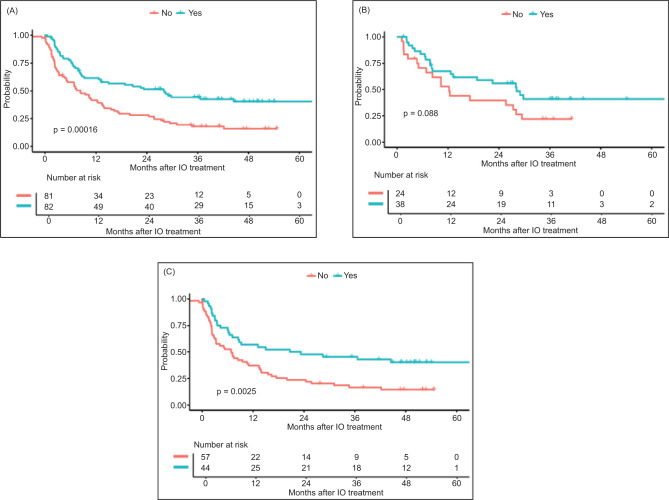
Progression-free survival (PFS) by clinical trial eligibility in (A) all patients; (B) axitinib/pembrolizumab cohort; and (C) ipilimumab/nivolumab cohort.

**Figure 2:**
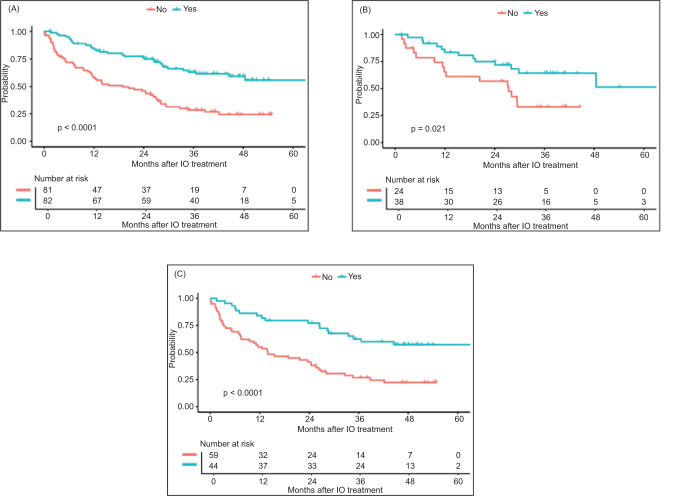
Overall survival (OS) by clinical trial eligibility in (A) all patients; (B) axitinib/pembrolizumab cohort; and (C) ipilimumab/nivolumab cohort.

The objective response rate (ORR) for the entire cohort (N = 165) was 42%, with an ORR of 52% and 31% for CTE and CTI patients, respectively. The median PFS for all patients was 12.62 months (95% CI 8.38–19.98) (Figure 1), and the median OS for the entire cohort was 29.37 months (95% CI 26.48–48.46) (Figure 2).

In the axitinib/pembrolizumab cohort, ORR for the entire cohort (n = 62) was 42%, mPFS 22.54 months (95% CI 12.22–29.77), and mOS 48.46 months (95% CI 27.99–NA). There was a numerically higher but not statistically significant difference in response rates between CTE and CTI patients (53% vs 25%; P = 0.11). The median PFS was numerically longer in CTE patients (28.12 vs 12.22 months; P = 0.09) (Table 3; Figure 1), and the median OS was higher in the CTE patients (not reached vs 27.37 months; P = 0.02) (Table 3; Figure 2). When excluding non-clear cell patients from the CTI patients, mPFS (12.22) and mOS (27.37) were unchanged.

**Table 3:** Outcomes based on clinical trial eligibility.

		Axitinib/Pembrolizumab				Ipilimumab/Nivolumab			
		All (n = 62)	Clinical trial eligible (n = 38)	Clinical trial ineligible (n = 24)	P	All (n = 103)	Clinical trial eligible (n = 44)	Clinical trial ineligible (n = 59)	P
PFS	Median months (95% CI)	22.54 (12.22, 29.77)	28.12 (13.21, NA)	12.22 (6.87, 29.44)	0.09	8.71 (6.41, 15.54)	21.98 (8.51, NA)	6.87 (3.15, 13.54)	0.003
	2-year rate (95% CI)	0.50 (0.38, 0.64)	0.56 (0.42, 0.75)	0.4 (0.24, 0.66)		0.34 (0.26, 0.44)	0.48 (0.35, 0.65)	0.24 (0.15, 0.37)	
	5year rate (95% CI)	0.33 (0.23, 0.49)	0.41 (0.27, 0.62)	NA		0.26 (0.18, 0.36)	0.40 (0.28, 0.58)	NA	
OS	Median months (95% CI)	48.46 (27.99, NA)	NA (29.80, NA)	27.37 (11.79, NA)	0.021	26.84 (21.68, 42.18)	71.49 (34.73, NA)	13.96 (9.49, 26.28)	< 0.0001
	2-year rate (95% CI)	0.68 (0.57, 0.81)	0.75 (0.62, 0.91)	0.57 (0.4, 0.81)		0.57 (0.48, 0.67)	0.77 (0.66, 0.91)	0.41 (0.30, 0.56)	
	5-year rate (95% CI)	0.41 (0.25, 0.69)	0.51 (0.31, 0.86)	NA		0.37 (0.29, 0.48)	0.57 (0.44, 0.75)	NA	

OS, overall survival; PFS, progression-free survival

In the ipilimumab/nivolumab cohort, ORR for the entire cohort (n = 103) was 42%, mPFS 8.71 months (95% CI 6.41–15.54), and mOS 26.84 months (95% CI 21.68–42.18). There was no statistically significant difference in response rates between CTE and CTI patients (45% vs 40%; P = 0.22). However, median PFS (21.98 vs 6.87 months; P = 0.003) and OS (71.49 vs 13.96 months; P < 0.0001) were higher in the CTE patients (Table 3; Figures 1 and 2). When excluding non-clear cell patients from the CTI patients, mPFS (7.2) and mOS (13.93) were not significantly changed.

A multivariable Cox model for OS and PFS by cohort was also performed (Table 4). Trial ineligible patients had significantly worse OS and PFS in the ipilimumab/nivolumab cohort, after adjusting for age, gender, and IMDC. However, the CTE status was not statistically significant for OS or PFS in axitinib/pembrolizumab cohort after adjusting for age, gender, and IMDC. The statistical power was limited for axitinib/pembrolizumab cohort due to smaller sample sizes and number of events.

**Table 4:** Multivariable analysis results of CTE versus CTI patients based on baseline characteristics.

Cohort	Endpoint	Factor	Comparison	Hazard ratio	95% LCL	95% UCL	P
Ipilimumab/ Nivolumab	**OS**	Trial Eligible	No vs Yes	3.564	1.922	6.607	<.0001
		Age at IO Start	≥ 60 vs <60	2.62	1.381	4.97	0.0032
		Gender	Female vs Male	1.139	0.646	2.007	0.6523
		IMDC	1–2 vs 0	1.247	0.645	2.411	0.5124
			3+ vs 0	1.968	0.908	4.267	0.0862
Ipilimumab/ Nivolumab	**PFS**	Trial eligible	No vs Yes	2.201	1.313	3.69	0.0028
		Age at IO start	≥60 vs <60	1.662	0.962	2.873	0.0687
		Gender	Female vs Male	1.04	0.606	1.784	0.8877
		IMDC	1–2 vs 0	0.825	0.455	1.498	0.5278
			3+ vs 0	1.335	0.661	2.693	0.4203
Axitinib/ Pembrolizumab	**OS**	Trial eligible	No vs Yes	2.046	0.91	4.601	0.0833
		Age at IO start	≥60 vs <60	1.754	0.649	4.741	0.268
		Gender	Female vs Male	0.964	0.404	2.299	0.9343
		IMDC	1–2 vs 0	1.223	0.459	3.259	0.6876
			3+ vs 0	2.792	0.877	8.888	0.0822
Axitinib/ Pembrolizumab	**PFS**	Trial eligible	No vs Yes	1.484	0.739	2.98	0.2668
		Age at IO start	≥60 vs <60	2.266	0.928	5.534	0.0725
		Gender	Female vs Male	0.872	0.423	1.799	0.7108
		IMDC	1–2 vs 0	1.207	0.537	2.712	0.6482
			3+ vs 0	2.902	1.074	7.838	0.0356

CTE, clinical trial eligible; CTI, clinical trial ineligible; IO, immune-oncology; OS, overall survival; PFS, progression-free survival.

## Discussion

The introduction of immunotherapy-based combinations for patients with treatment-naïve mRCC has revolutionized the care of these patients and significantly improved OS. However, the restrictive inclusion and exclusion criteria for these trials result in clinical outcomes that may not be reproducible in the real-world setting (6, 7). In this analysis, patients with treatment-naïve mRCC treated with ipilimumab/nivolumab or axitinib/pembrolizumab who would not have met their respective registration clinical trial eligibility criteria had worse outcomes than those who would have met clinical trial criteria. The percentage of patients who would have been ineligible for the registration trials was relatively high for both axitinib/pembrolizumab and ipilimumab/nivolumab cohorts, at 39% and 57%, respectively.

There are two primary consequences to the overly restrictive eligibility criteria in clinical trials. First, they limit enrolment to clinical trials, which delays potential advances in drug development and approval. The second concern is that limiting clinical trial enrolment based on strict eligibility criteria may be self-selective for a generally healthier patient population that does not reflect the real-world population to which these therapies will ultimately be applied.

These concerns have resulted in ongoing efforts to relax clinical trial eligibility and broaden the population who may subsequently participate in clinical trials. The American Society of Clinical Oncology (ASCO) and Friends of Cancer Research established working groups that proposed several recommendations to make clinical trials more representative and inclusive (9). In broad terms, the proposal recommends only excluding patients from clinical trials if there is a compelling scientific rationale or evidence that a patient’s safety would be compromised by enrollment, inclusion and exclusion criteria be tailored to the study objectives, and that the study population more closely resemble the real-world populations without excluding specific groups in the absence of scientific justification. More specifically, this working group provided detailed recommendations on how to improve eligibility criteria such as duration of washout periods, prior treatments, laboratory testing periods and reference ranges, and performance status (9).

It is important to note that an obvious motivating factor for stringent eligibility criteria is a concern about patient safety in clinical trials, and it is critical to carefully select the healthiest patients and those with the fewest medical comorbidities. However, the US Food and Drug Administration (FDA) conducted a review of eligibility criteria for clinical trials submitted as investigational new drug (IND) applications to the FDA Office of Hematology and Oncology Drug Products (OHOP) in 2015. A total of 297 oncology trials were reviewed. A primary conclusion of this analysis was that eligibility criteria are overly restrictive and that expanding these criteria can be done without negatively impacting patient safety (8).

Based on these findings, the FDA produced multiple draft guidance documents for industry and Institutional Review Boards (IRBs) on cancer clinical trial eligibility criteria. More specifically, the guidance revolved around eligibility criteria for patients with brain metastases, eligibility of patients with organ dysfunction or prior or concurrent malignancies, and guidance for inclusion of patients with HIV, hepatitis B virus, or hepatitis C virus infections (10–12). Draft recommendations are also being developed as guidance for the eligibility criteria related to washout periods, concomitant medications, and performance status (13, 14). The importance of these areas of focus is highlighted in the present report, as lab abnormalities and brain metastases were two of the three most common reasons for clinical trial ineligibility in both axitinib/pembrolizumab and ipilimumab/nivolumab cohorts (Table 2).

There are inherent limitations to the findings described in this report. This study’s findings are of patients treated at a single academic institution and may not be reflective of a broader patient population. The analysis was also only limited to patients treated with ipilimumab/nivolumab or axitinib/pembrolizumab. Although these findings may be reproducible in other mRCC treatments as well, those analyses need to be completed. Despite these limitations, the data highlight the discrepant outcomes between patients who would meet trial eligibility and those who would not.

## Conclusion

A high percentage of real-world patients are ineligible for RCC clinical trials, and these patients have worse PFS and OS when compared with those who are trial-eligible. Additional treatment options as well as strategies are needed for these patients to include them in prospective trials. Developments are ongoing to broaden clinical trial eligibility to improve trial inclusivity.
